# Fracture of the Dislocated Humerus

**Published:** 1894-06

**Authors:** 


FRACTURE OF THE DISLOCATED HUMERUS.—Dr. Charles McBurney has recently treated a case of sub-coracoid dislocation of the humerus, complicated by a fracture of the neck, in a novel and suggestive manner. Several surgeons having tried, under ether narcosis, to effect reduction of the head of the bone, and Dr. McBurney himself having made the effort, a heavy bone drill was ordered from the instrument maker, together with a large, blunt, right-angled hook which fitted well into the opening made by the drill, which was provided with a strong transverse handle. Two weeks after the injury was received an incision was made over the head of the humerus, a hole was drilled in the head of the bone and traction applied downward and outward. The head of the bone was thus restored to its normal position with relative ease. The deep parts were united with catgut and the superficial wound closed. Treatment by rest and fixation was wholly satisfactory; the bone united firmly; the joint structures healed smoothly and the function was perfectly restored.

The method used in this case is full of suggestiveness for the treatment of fractures of other bones, complicated with dislocation, especially of the femur.—*Annals of Surgery*, April, 1894, *Ex.*

